# Compilation and evaluation of gas phase diffusion coefficients of halogenated organic compounds

**DOI:** 10.1098/rsos.171936

**Published:** 2018-07-18

**Authors:** Wenjun Gu, Peng Cheng, Mingjin Tang

**Affiliations:** 1 State Key Laboratory of Organic Geochemistry and Guangdong Key Laboratory of Environmental Protection and Resources Utilization, Guangzhou Institute of Geochemistry, Chinese Academy of Sciences, Guangzhou 510640, People's Republic of China; 2 University of Chinese Academy of Sciences, Beijing 100049, People's Republic of China; 3 Institute of Mass Spectrometer and Atmospheric Environment and Guangdong Provincial Engineering Research Center for on-line source apportionment system of air pollution, Jinan University, Guangzhou 510632, People's Republic of China

**Keywords:** organic halogens, gas phase diffusion, mean free path

## Abstract

Organic halogens are of great environmental and climatic concern. In this work, we have compiled their gas phase diffusivities (pressure-normalized diffusion coefficients) in a variety of bath gases experimentally measured by previous studies. It is found that diffusivities estimated using Fuller's semi-empirical method agree very well with measured values for organic halogens. In addition, we find that at a given temperature and pressure, different molecules exhibit very similar mean free paths in the same bath gas, and then propose a method to estimate mean free paths in different bath gases. For example, the pressure-normalized mean free paths are estimated to be 90, 350, 90, 80, 120 nm atm in air (and N_2_/O_2_), He, argon, CO_2_ and CH_4_, respectively, with estimated errors of around ±25%. A generic method, which requires less input parameter than Fuller's method, is proposed to calculate gas phase diffusivities. We find that gas phase diffusivities in He (and air as well) calculated using our method show fairly good agreement with those measured experimentally and estimated using Fuller's method. Our method is particularly useful for the estimation of gas phase diffusivities when the trace gas contains atoms whose diffusion volumes are not known.

## 1. Introduction

Gas–surface interactions play important roles in many aspects of atmospheric chemistry and physics, including heterogeneous and multiphase reactions of atmospheric aerosol particles, cloud and rain droplets, and ice particles [1–4]. Atmosphere–ocean and atmosphere–land exchanges of gas molecules, such as dry deposition of trace gases, can also be considered as gas–surface interactions [5–7]. Recently, interactions with surfaces of human-made structures have been proposed as potentially important sources/sinks for several reactive trace gases [8–11]. The importance of gas–surface interactions has also been widely recognized in other fundamental and applied research areas, such as heterogeneous catalysis [12–14].

Gas–surface interactions typically consist of coupled physical and chemical processes in and between different phases [15–17]. Diffusion of gas molecules towards the surface is the first step for their uptake by the surface [16], and vice versa it is the final step for the release of gas molecules from the surface. The rate of gas phase diffusion depends on the concentration gradient and the gas phase diffusion coefficient [18], and thus knowledge of gas phase diffusion coefficients is critical for understanding gas–surface interactions. However, until recently not much attention has been paid to gas phase diffusion coefficients. Only a few previous studies have collected gas phase diffusion coefficients reported in the literature in order to assess the performance of theoretical calculations [19–22], and the number of molecules included in those studies is quite limited. Gordon [23] compiled a comprehensive list of references which reported experimentally measured gas phase diffusion coefficients; however, no data have been provided in this report [23].

In our previous work, we have compiled and evaluated gas phase diffusion coefficients of inorganic reactive trace gases in different bath gases [16] and organic reactive trace gases (containing no halogen atoms) in air (and N_2_/O_2_) [24]. We found that Fuller's semi-empirical method [22] could reliably estimate gas phase diffusivities (pressure-normalized diffusion coefficients), and differences between measured and estimated diffusivities using Fuller's method are typically less than 30% for inorganic compounds [16] and less than 10% for organic compounds [24]. It is further found that although diffusivities of different molecules in air vary largely, their Knudsen numbers at a given pressure are almost constant for a given particle size. This is because their mean free paths in air show little variation for different molecules. Expanding upon our previous studies [16,24], in this work, we have compiled experimentally measured gas phase diffusivities of organic halogens in a variety of bath gases reported in the literature. Organic halogens are of great environmental and climatic concern because they can be stratospheric ozone depletion species [25], greenhouse gases [26] and harmful pollutants [27–29]. It is found that measured diffusivities of organic halogens agree well with estimated values using Fuller's method. In addition, we propose a method based on mean free paths to calculate gas phase diffusivities. At a given temperature, this method only requires the average molecular speed (essentially only depending on its molecular mass) and the mean free path in the bath gas as input parameters. We have shown that the calculated diffusivities using our method agree quite well with measured values and those estimated using Fuller's method. Our method is particularly useful if the diffusion volumes of molecules of interest are unknown, i.e. when Fuller's method cannot be used due to lack of input parameters.

## 2. Theoretical background

The diffusion of molecules in fluids is driven by their concentration gradient, as described by Fick's first law [18]:

2.1*a* $$J=D^{*}\cdot \frac{\partial n}{\partial x},$$

where *J* is the flux of molecules along the *x*-axis (molecule cm^−2^ s^−1^), and D* is the diffusion coefficient (cm^2^ s^−1^), *n* is the concentration of molecules (molecule cm^−3^) and *x* is the distance along the *x*-axis. Diffusion causes the concentration to change with time, given by Fick's second law [18]:

2.1*b* $$\frac{\partial n}{\partial t}=D^{*}\cdot \frac{\partial^{2}n}{\partial x^{2}},$$

where *t* is the time (s). Equations (2.1*a*) and (2.1*b*) only consider diffusion along one dimension, and equations for diffusion in three dimensions can be found elsewhere [30]. It should be pointed out that typically *D*, instead of D*, is used for the diffusion coefficient. However, in this work, *D* has been reserved for the diffusivity (or pressure-independent diffusion coefficient) in order to be consistent with our previous work [16,24] as detailed below.

The gas phase diffusion coefficient, *D*_P_, depends on the pressure of the bath gas (*P*, in the unit of Torr) and is related to the diffusivity (or pressure-independent diffusion coefficient), *D* (Torr cm^2^ s^−1^), by the following equation [16]:

2.2 $$D=D_{P}\cdot P.$$

Gas phase diffusivities, when experimental data are not available, can be estimated using a variety of methods which vary in complexity and performance. Reid *et al*. [20] compared measured gas phase diffusivities of a large body of compounds with estimated values using several widely adopted methods. It was concluded that on average the semi-empirical method developed by Fuller *et al*. [21,22] shows the best performance. Fuller's method has also been used in our previous work to estimate gas phase diffusivities of inorganic compounds and organic compounds which do not contain halogen atoms [16,24], and in general good agreement of estimated values with experimental data has been found.

According to Fuller's method, the diffusivity of a trace gas *X* in a bath gas *A* can be calculated by the following equation:

2.3*a* $$D(X,A)=\frac{1.0868\cdot T^{1.75}}{\sqrt{m(X,A)}\cdot {\left(\sqrt[{3}]{V_{X}}+\sqrt[{3}]{V_{A}}\right)}^{2}},$$

where *D*(*X*,*A*) is the gas phase diffusivity of *X* in *A* (Torr cm^2^ s^−1^), *T* is the temperature (K), *m*(*X*,*A*) is the reduced mass of the molecular pair *X-A* (g mol^−1^), and *V_X_* and *V_A_* are the dimensionless diffusion volumes of *X* and *A*, respectively. The reduced molecular mass, *m*(*X,A*), can be calculated by the following equation:

2.3*b* $$m(X,A)=\frac{2}{\left(1/m_{X}+1/m_{A}\right)},$$

where *m_X_* and *m_A_* are the molar masses (g mo1^−1^) of *X* and *A*, respectively. The diffusion volume of a molecule can be derived from the atomic diffusion volumes of atoms it contains, given by the following equation:

2.3*c* $$V=\sum^{n_{i}}\cdot V_{i},$$

where *n_i_* is the number of the atom with a diffusion volume of *V_i_*.

Table 1 lists atomic and structural diffusion volumes of common atoms and structures. In addition, the diffusion volumes of some small molecules, e.g. N_2_, H_2_O and CO_2_, are also given in table 1. If the diffusion volume of a molecule can be found in table 1, calculation of its diffusion volume using equation (2.3*c*) is unnecessary. The measured and estimated diffusivities in air are very close to those in N_2_ and O_2_ [24]. Therefore, as done in our previous study [24], we did not differentiate measured diffusivities in air, N_2_ and O_2_, and estimated diffusivities in N_2_ and O_2_ were also assumed to be equal to those in air. More details of description and discussion of Fuller's method can be found elsewhere [16,20,24].

**Table 1.** Dimensionless diffusion volumes (*V*) of common atoms, structures and small molecules [20].

species	C	H	O	N	S	aromatic ring
*V*	15.9	2.31	6.11	4.54	22.9	−18.3
species	F	Cl	Br	I		heterocyclic ring
*V*	14.7	21	21.9	29.8		−18.3
species	He	Ne	Ar	Kr	Xe	H_2_
*V*	2.67	5.98	16.2	24.5	32.7	6.12
species	D_2_	N_2_	O_2_	air	CO	CO_2_
*V*	6.84	18.5	16.3	19.7	18	26.9
species	NH_3_	H_2_O	SF_6_	SO_2_	Cl_2_	Br_2_
*V*	20.7	13.1	71.3	41.8	38.4	69

## 3. Results

**Figure 1.**
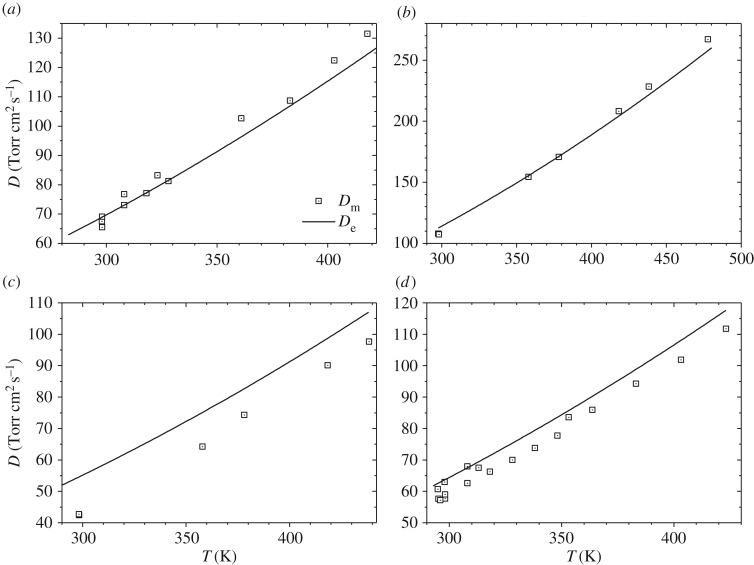
Comparison of measured (*D*_m_) and estimated diffusivities (*D*_e_) as a function of temperature [31–39]. (*a*) CHCl_3_ in air, (*b*) CH_3_Cl in CH_4_, (*c*) CH_2_CH_3_Cl in CH_3_Cl and (*d*) CCl_4_ in air.

The measured gas phase diffusivities of 61 halogenated organic compounds in a variety of bath gases (including N_2_, air, H_2_, noble gases and some organic compounds) at different temperatures, reported by previous studies, are summarized in the electronic supplementary material together with corresponding estimated diffusivities using Fuller's method. This electronic supplementary material has been prepared in a similar format to that used in our previous work [16,24]. In addition, figure 1 displays measured and estimated diffusivities of CHCl_3_ in air, CH_3_Cl in CH_4_, CH_3_CH_2_Cl in CH_3_Cl and CCl_4_ in air at different temperatures as examples.

As evident from data contained in the electronic supplementary material and displayed in figure 1, measured diffusivities show good agreement with estimated values. Differences between the measured and estimated diffusivities are smaller than 10% for most of the data, and it is very rare for the differences to exceed 20%. It should also be noted that the entire dataset covers a wide temperature range from 273 to 478 K. Therefore, it can be concluded that Fuller's method can reliably estimate gas phase diffusivities of halogenated organic compounds as a function of temperature. Similar conclusions have been drawn in our previous work for inorganic compounds [16] and organic compounds which do not contain halogen atoms [24].

## 4. Discussion

An interesting question has been raised: can we estimate its diffusivity if the molecule contains atoms with diffusion volumes being unknown (i.e. not listed in table 1)? In our previous work [24], it has been found that for a given particle diameter, the Knudsen number (*Kn*), which is the ratio of the mean free path of gas molecules to the particle radius, is identical for different trace gases with very different diffusion coefficients. This is because different trace gases have almost the same molecule mean free paths in a given bath gas. The mean free path depends on pressure and temperature, and the relation between the pressure-normalized mean free path and the diffusion coefficient is given by the following equations [18,24]:

4.1*a* $$D_{P}(X,A)=\frac{\lambda_{P}(A)\cdot c(X)}{3}$$

and

4.1*b* $$\lambda_{P}(A)=\frac{3D_{P}(X,A)}{c(X)},$$

where *D*_P_(*X*,*A*) is the diffusivity (atm cm^2^ s^−1^) of *X* in *A*, *λ*_P_(*A*) is the pressure-normalized mean free path (nm atm) in *A* and *c*(*X*) is the average molecular speed of *X* (cm s^−1^).

Our previous work [24] has shown that *λ*_P_ for air (and N_2_ and O_2_) calculated from diffusivities (measured or estimated using Fuller's method) and average molecular speeds of different molecules using equation (4.1*b*) are essentially identical. The mean free path in 1 atm air at 298 K is estimated to be 100 ± 20 nm [24]. Careful examination of fig. 3 in the paper published by Tang *et al*. [24] reveals that a value of approximately 90 nm may be more proper in general, especially for organic molecules. Within uncertainties, this shows fairly good agreement with the mean free path in air (approx. 67 nm in 1 atm air at 298 K) derived from viscosity data for air [40]. One reason to explain the difference in the mean free paths derived from diffusivities and viscosities is that the underlying assumption (made by classical gas kinetic theory) that gas molecules behave as hard spheres is not strictly valid. Compared to those derived from viscosity data, mean free paths derived from diffusivities data should be more accurate for the estimation of gas phase diffusivities.

**Figure 2.**
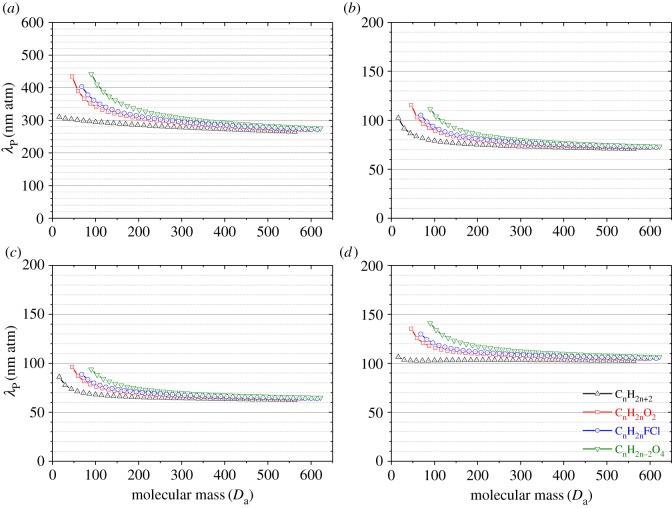
Calculated pressure-normalized mean free paths in (*a*) He, (*b*) argon, (*c*) CO_2_ and (*d*) CH_4_ at 298 K as a function of molecular masses using equation (4.1*b*). Diffusivities are estimated using Fuller's methods for C_n_H_2n+2_ (alkanes), C_n_H_2n_O_2_ (monocarboxylic acid), C_n_H_2n_FCl (alkanes with two hydrogen atoms replaced by one F atom and one Cl atom) and C_n_H_2n−2_O_4_ (dicarboxylic acid) molecules with carbon atom numbers (*n*) ranging from 1 to 20.

Using a similar procedure to Tang *et al*. [24], we first estimate diffusivities of four different groups of organic compounds (alkanes, alkanes with two hydrogen atoms replaced by one F atom and one Cl atom, monocarboxylic acids and dicarboxylic acids) in He, argon, CO_2_ and CH_4_ at 298 K using Fuller's method, and then calculate *λ*_P_ in He, argon, CO_2_ and CH_4_ using equation (4.1*b*). Figure 2 shows the results, suggesting that calculated *λ*_P_ are very similar for different trace gases in the same bath gas. The pressure-normalized mean free paths at 298 K, estimated using diffusivities, are calculated to be 350, 90, 80, 120 nm atm in He, argon, CO_2_ and CH_4_, respectively, and their uncertainties are estimated to be around ±25%. Pressure-normalized mean free paths can also be derived for other bath gases using the same approach as long as their diffusion volumes can be calculated using the atomic diffusion volumes given in table 1.

Diffusivities can then be calculated from mean free paths, using equation (4.1*a*). Our previous work [24] implies that diffusivities of organic molecules (which do not contain halogen atoms) in air (and N_2_/O_2_) calculated using equation (4.1*a*) show good agreement with measured values and those estimated using Fuller's method. In our current work, we compare diffusivities in He at 298 K calculated using equation (4.1*a*) with those measured and estimated using Fuller's method, in order to further verify the applicability and reliability of equation (4.1*a*). He is chosen here because it has been widely used as bath gas in measurements of gas phase diffusion coefficients. If measurements were not carried out at 298 K, reported values have been extrapolated to those at 298 K using the temperature dependence given by the following equation [16]:

4.2 $$D(298\;K)=D(T)\cdot {\left(\frac{298\;\text{K}}{T}\right)}^{1.75}$$

where *D*(*T*) and *D*(298 K) are the measured diffusivity at *T* (in K) and the extrapolated value at 298 K, respectively. If one study reported diffusivities at several temperatures, the measurement carried out at the temperature closed to 298 K is used.

Tables 2 and 3 list measured diffusivities (*D*_m_) and those estimated using Fuller's method (*D*_e_) and calculated using equation (4.1*a*) (*D*_c_). It can be concluded that diffusivities calculated using equation (4.1*a*) agree fairly well with measured values. The differences between *D*_c_ and *D*_m_ are mostly within 30% for inorganic species (table 1) and within 20% for organic halogens (table 2). We note that overall the difference between *D*_c_ and *D*_m_ is larger than that between *D*_e_ and *D*_m_, i.e. the performance of Fuller's method is better. However, estimation of diffusivities using Fuller's method requires the knowledge of molecular mass and diffusion volumes; if a molecule contains atoms whose diffusion volumes are not known (i.e. not listed in table 1), Fuller's method cannot be used. Therefore, although Fuller's method appears to be more accurate, equation (4.1*a*) provides a simpler and more generalized method which requires fewer parameters as input. This is particularly useful for molecules containing atoms whose diffusion volumes are not known. It should be pointed out that there are a few other methods which can be used to calculate the gas phase diffusivities [20], such as the Lennard–Jones method. However, the majority of existing methods require molecular parameters which are not readily available and can only be derived from molecular dynamics simulations. Reid *et al*. [20] evaluated the performance of several widely used methods and concluded that Fuller's method, though simpler than other methods, in general generates the smallest errors when compared with experimental data.

**Table 2.** Comparison of diffusivities (Torr cm^2^ s^−1^) experimentally measured by previous work (*D*_m_), estimated using Fuller's method (*D*_e_), and calculated (*D*_c_) using equation (4.1*a*) for inorganic compounds at 298 K.

molecule	*D*_m_	*D*_e_	*D*_c_	*D*_e_/*D*_m_	*D*_c_/*D*_m_	reference
NO_3_	402	595	283	1.48	0.70	[41]
HONO	443	519	325	1.17	0.73	[42]
	484	519	325	1.07	0.67	[43]
O_3_	415	528	322	1.27	0.78	[44]
OH	671	779	540	1.16	0.81	[44]
	670	779	540	1.16	0.81	[45]
	622	780	540	1.25	0.87	[46]
HO_2_	410	594	388	1.45	0.95	[44]
	435	594	388	1.37	0.89	[46]
HOBr	369	412	226	1.12	0.61	[47]
	311	412	226	1.32	0.73	[48]
HOI	340	368	186	1.08	0.55	[49]
ClNO_2_	317	374	247	1.18	0.78	[50]
ICl	302	320	175	1.06	0.58	[51]

**Table 3.** Comparison of diffusivities (Torr cm^2^ s^−1^) experimentally measured by previous work (*D*_m_), estimated using Fuller's method (*D*_e_) and calculated (*D*_c_) using equation (4.1*a*) for organic halogens at 298 K.

gas	*D*_m_	*D*_e_	*D*_c_	*D*_e_/*D*_m_	*D*_c_/*D*_m_
CH_3_I	312	316	187	1.01	0.60
CH_2_F_2_	348	332	309	0.95	0.89
CH_2_Cl_2_	303	293	242	0.97	0.80
CH_2_Br_2_	268	292	169	1.09	0.63
CHCl_3_	251	255	204	1.02	0.81
CH_3_CH_2_Br	298	291	213	0.98	0.72
CH_3_CH_2_I	261	269	178	1.03	0.68
CH_3_CHF_2_	302	277	274	0.92	0.91
CH_2_ClCH_2_Cl	277	254	224	0.92	0.81
CH_3_CH_2_CH_2_Cl	255	252	252	0.99	0.99
CH_3_CH_2_CH_2_Br	239	252	201	1.06	0.84
CH_3_CHBrCH_3_	245	252	201	1.03	0.82
CH_3_CH_2_CH_2_I	232	236	171	1.02	0.74
CH_3_CHICH_3_	232	236	171	1.02	0.74
CH_3_CHBrCH_2_Cl	231	225	178	0.98	0.77
1-chlorobutane	223	225	232	1.01	1.04
2-chlorobutane	225	225	232	1.00	1.03
1-bromobutane	221	224	190	1.02	0.86
2-bromobutane	224	224	190	1.00	0.85
1-iodobutane	211	213	164	1.01	0.78
2-iodobutane	221	213	164	0.97	0.74
1-chloropentane	209	204	216	0.98	1.03
1-fluorohexane	195	193	164	0.99	0.84
1-bromohexane	186	188	173	1.01	0.93
2-bromohexane	189	188	173	0.99	0.92
3-bromohexane	188	188	173	1.00	0.92
fluorobenzene	226	208	227	0.92	1.00
chlorobenzene	216	202	210	0.94	0.97
bromobenzene	220	202	178	0.92	0.81
hexafluorobenzene	182	166	163	0.91	0.90
4-fluorotoluene	202	191	212	0.95	1.05

## 5. Conclusion

In this work, we have compiled gas phase diffusivities (pressure-independent diffusion coefficients) of organic halogens in a variety of bath gases. It is found that estimated diffusivities using Fuller's semi-empirical method agree very well with experimental values reported in the literature, suggesting that Fuller's method can reliably estimate gas phase diffusivities of organic halogens. We also find that in addition to air (and N_2_/O_2_), within uncertainties the mean free paths of molecules are almost identical for a given bath gas. The pressure-normalized mean free paths at 298 K are estimated to be 90, 350, 90, 80, 120 nm atm in air (and N_2_/O_2_), He, argon, CO_2_ and CH_4_, respectively, with estimated errors of around ±25%. The pressure-normalized mean free paths in other bath gases, as long as diffusion volumes of bath gases can be calculated using the atomic volumes listed in table 1, can be derived using the same approach.

We further propose a generic method, based on mean free paths, to calculate gas phase diffusivities. Our proposed method only requires the mean free path in the bath gas and the average molecular speed of the trace gas (only depending on its molecular mass) as input parameters. The mean free path in a bath gas can be derived using the approach described in §4 as long as the diffusion volume of the bath gas can be calculated using atomic and structural diffusion volumes listed in table 1. Calculated diffusivities in He (and air/N_2_/O_2_ as well) using our method show reasonably good agreement with measured values and those estimated using Fuller's method. Our method is particularly useful when a trace gas contains atoms whose diffusion volumes are not known (i.e. when Fuller's method cannot be applied due to lack of input parameters).

## Supplementary Material

Compilation and evaluation of diffusivities

## Data accessibility

The datasets used by this article have been uploaded as electronic supplementary material.
