# Supplementary material for: Compilation and evaluation of gas phase diffusion coefficients of halogenated organic compounds
Source: R Soc Open Sci. 2018 Jul 18;5(7):171936. doi: 10.1098/rsos.171936 (PMC6083652; doi:10.1098/rsos.171936)
Supplement: Compilation and evaluation of diffusivities [file rsos171936supp1.pdf]

Supporting information for “Compilation and evaluation of gas phase diffusion coefficients of halogenated organic compounds”

Instruction:

1. Units used in this work is K for temperature (T) and Torr for pressure (P).

2. Experimentally measured diffusion coefficients reported in literature have been converted to measured diffusivities (D_m) in the unit of Torr cm^2 s^{-1}, and Fuller’s semi-empirical method is used in this work to estimate diffusivities (D_e, in the unit of Torr cm^2 s^{-1}).

Table of Contents

1 Compounds with one carbon atom 1

1.1 CH_3Cl 1

1.2 CH_3I 3

1.3 CH_2F_2 4

1.4 CH_2Cl_2 5

1.5 CH_2ClBr 7

1.6 CH_2Br_2 8

1.7 $CHCl_3$ 9

1.8 $CHBr_3$ 11

1.9 CF_2Cl_2 12

1.10 CF_4 13

1.11 CCl_4 16

1.12 CCl_2O, CCl_3NO_2, and CNCl 19

2 Compounds with two carbon atoms 20

2.1 CH_3CH_2Cl 20

2.2 CH_3CH_2Br 21

2.3 CH_3CH_2I 22

2.4 CH_3CHF_2 23

2.5 CH_2ClCH_2Cl 24

2.6 CH_2BrCH_2Br 25

2.7 CCl_3CH_3 and $CHCl_2CH_2Cl$ 26

2.8 $CHCl_2CHCl_2$, CCl_3CHCl_2, and CH_2ClCH_2OH 27

2.9 CH_2=CHCl, CHCl=CHCl, CHCl=CCl_2, and CCl_2=CCl_2 28

3 Compounds with three or more carbon atoms 29

3.1 $CH_3CH_2CH_2Cl$, $CH_3CH_2CH_2Br$, and $CH_3CHBrCH_3$ 29

3.2 $CH_3CH_2CH_2I$ and CH_3CHICH_3 30

3.3 $CH_3CHClCH_2Cl$, CH_3CHBrCH2Cl, $CH_3BrCHBrCH_2Cl$, and CH_2=$CHCH_2Cl$. 31

3.4 1-chlorobutane and 2-chlorobutane 32

3.5 1–bromobutane and 2-bromobutane 33

3.6 1-iodobutane, 2-iodobutane, and dichloroethyl ether 34

3.7 1-chloropentane and 1-bromo-3-methyl-butane 35

3.8 1-fluorohexane, 1-bromohexane, 2-bromohexane, and 3-bromohexane 36

3.9 C_7F_{18} 37
4 Aromatic compounds 38
4.1 fluorobenzene, chlorobenzene, and bromobenzene 38
4.2 hexafluorobenzene 40
4.3 4-fluorotoluene, 2-chlorotoluene, 3-chlorotoluene, 4-chlorotoluene, and benzyl chloride 41

1 Compounds with one carbon atom

1.1 CH_3Cl

bath gas	reference.	*T*	*P*	*D*m	*D*e	(*D*m-*D*e)/*D*e (%)
air	[1]	298	760	109±2	99	10
CH_4	[2]	298	740	108	113	-4
		358	739	154	156	-1
		418	745	208	205	2
		478	735	267	258	3
		298	739	107	113	-5
		378	743	171	172	-1
		438	733	228	222	3
SO_2	[3]	303	760	53	65	-19
		313	760	56	69	-19
		323	760	58	73	-20
		333	760	63	77	-18
CH_3OCH_3	[3]	303	760	51	66	-21
		313	760	55	69	-21
		323	760	57	73	-22
		333	760	62	77	-20

The diffusivity of CH_3Cl in air at 298 K was measured by Cowie and Watts,[1] and the measured diffusivity is 10% larger than the estimated value.

Gotoh et al. measured the diffisitivities of CH_3Cl in CH_4 from 298 to 438 K,[2] and the differences between the measured and estimated diffusivities are <5% across the entire temperature range.

The diffusitivities of CH_3Cl in SO_2 and CH_3OCH_3, measured by Chakraborti and Gray,[3] are around 20% smaller than estimated values for temperatures in the range of 303 to 333 K.

References:

[1] Cowie, M., and Watts, H.: Diffusion of methane and chloromethanes in air, ***Canadian Journal of Chemistry,*** 49, 74-77, 1971.

[2] Gotoh, S., Manner, M., Sorensen, J. P., and Stewart, W. E.: Binary diffusion-coefficients of low-density gases .1. Measurements by modifide loschmidt method, ***J. Chem. Eng. Data***, 19, 169-171, 1974.

[3] Chakraborti, P. K., and Gray, P.: Diffusion coefficients in binary mixtures of polar gas-sulphur dioxide, dimethtl ether and methyl chlorine, ***Transactions of the Faraday Society***, 62, 3331-3337, 1966.

1.2 CH_3I

bath gas	reference	*T*	*P*	D_m	D_e	$(D_m-D_e)/D_e$ (%)
He	[1]	431	760	595±12	602	-1

The diffusivity of CH_3I in He, measured at 431 K by Fuller et al.,[1] is only 1% smaller than the estimated value.

References:

[1] Fuller, E. N., Ensley, K., and Giddings, J. C.: Diffusion of halogenated hydrocarbons in helium. Effect of structure on collision cross sections, ***J. Phys. Chem.***, 73, 3679-3685, 1969.

1.3 CH_2F_2

bath gas	reference	*T*	*P*	D_m	D_e	$(D_m-D_e)/D_e$ (%)
He	[1]	431	760	664±23	633	5

The diffusivity of CH_2F_2 in He, measured at 431 K by Fuller et al.,[1] is 5% laregr than the estimated value.

References:

[1] Fuller, E. N., Ensley, K., and Giddings, J. C.: Diffusion of halogenated hydrocarbons in helium. Effect of structure on collision cross sections, ***J. Phys. Chem.***, 73, 3679-3685, 1969.

1.4 CH_2Cl_2

bath gas	reference	T	P	D_m	D_e	$(D_m-D_e)/D_e$ (%)
He	[1]	428	760	570±7	552	3
air	[2]	298	760	79±4	79	0
	[3]	298	760	79±4	79	-1
	[4]	288	760	76	75	1
		298	760	79	80	0
		308	760	83	84	-1
Kr	[5]	278	16	39	48	-17
		288	18	42	50	-17
		303	21	47	55	-15
		318	24	51	60	-15

The diffusivity of CH_2Cl_2 in He at 428 K, measured Fuller et al.,[1] is 3% larger than the estimated value.

The diffusivities of CH_2Cl_2 in air, measured at 298 K by Cowie and Watts [2]] and by Lugg [3] and from 288 to 308 K by Watts,[4] show excellent agreement with estimated values, with difference being 1% or smaller.

The diffusivities of CH_2Cl_2 in Kr, measured by Singh and Srivastava from 278 to 318 K,[5] are 15% to 17% smaller than the estimated values.

References:

[1] Fuller, E. N., Ensley, K., and Giddings, J. C.: Diffusion of halogenated hydrocarbons in helium. Effect of structure on collision cross sections, ***J. Phys. Chem.***, 73, 3679-3685, 1969.

[2] Cowie, M., and Watts, H.: Diffusion of methane and chloromethanes in air, ***Canadian Journal of Chemistry***, 49, 74-77, 1971.

[3] Lugg, G. A.: Diffusion coefficients of some organic and other vapors in air, ***Analytical Chemistry***, 40, 1072-1077, 1968.

[4] Watts, H.: Temperature dependence of diffusion of carbon tetrachloride, chloroform and methylene chloride vapors in air by a rate of evaporation method, ***Canadian Journal of Chemistry***, 49, 67-73, 1971.

[5], B. N.: Unlike interactions and binary diffusion in polar-nonpolar mixtures-krypton-methylene chloride and krypton-ethylchloride, ***International Journal of Heat and Mass Transfer***, 11, 1771-1778, 1968.

1.5 CH_2ClBr

bath gas	reference	*T*	*P*	D_m	D_e	$(D_m-D_e)/D_e$ (%)
air	[1]	298	760	72±1	81	-10

The measured diffusivity of CH_2ClBr in air at 298 K [1] is 10% smaller than the estimated value.

References:

[1] Lugg, G. A.: Diffusion coefficients of some organic and other vapors in air, ***Analytical Chemistry***, 40, 1072-1077, 1968.

1.6 CH_2Br_2

bath gas	reference	*T*	*P*	D_m	D_e	$(D_m-D_e)/D_e$ (%)
He	[1]	428	760	505±5	550	-8

The measured diffusivity of CH_2Br_2 in He at 428 K [1] is 8% smaller than the estimated value.

References:

[1] Fuller, E. N., Ensley, K., and Giddings, J. C.: Diffusion of halogenated hydrocarbons in helium. Effect of structure on collision cross sections, ***J. Phys. Chem.***, 73, 3679-3685, 1969.

1.7 $CHCl_3$

bath gas	reference	T	P	D_m	D_e	$(D_m-D_e)/D_e$ (%)
He	[1]	429	760	474±9	483	-2
N_2	[2]	361	760	103	99	3
		383	760	109	110	-1
		403	760	122	120	2
		418	760	131	128	3
air	[3]	308	760	77	73	5
	[4]	298	760	67±1	69	-2
	[5]	323	760	83±2	79	5
	[6]	298	760	66±1	69	-5
	[7]	298	760	69	69	0
		308	760	73	73	0
		318	760	77	77	0
		328	760	81	81	0
CO_2	[2]	363	760	84	76	10
		383	760	91	84	9
		404	760	98	92	7
Kr	[8]	284	11	36	41	-12
		293	11	38	43	-13
		303	13	41	46	-12
		313	13	43	49	-12
$C_2H_5OC_2H_5$	[9]	293	760	22	30	-25

The diffusivity of $CHCl_3$ in He at 429 K, measured by Fuller et al.,[1] is 2% smaller than the estimated value.

Nagata and Hasegawa investigated the temperature dependence of diffusivities of $CHCl_3$ in N_2 from 361 to 418 K,[2] and differences between the measured and estimated values are not larger than 3%.

Five studies measured the diffusivities of $CHCl_3$ in air at different temperatures from 298 to 328 K.[3-7] Differences between the measured and estimated diffusivities are around 5% or smaller.

Nagata and Hasegawa measured the diffusivities of $CHCl_3$ in CO_2 at 363, 383, and 404 K.[2] The differences between the measured and estimated diffusivities are around 10% or smaller.

Srivastava and Saran measured the diffusivities of $CHCl_3$ in Kr at 284, 293, 303, and 313 K,[8] and the measured diffusivities are around 13% smaller than the estimated values.

The diffusivity of $CHCl_3$ in $C_2H_5OC_2H_5$ at 293 K, measured by Weissman,[9] is 25% smaller than the estimated value.

References:

[1] Fuller, E. N., Ensley, K., and Giddings, J. C.: Diffusion of halogenated hydrocarbons in helium. Effect of structure on collision cross sections, ***J. Phys. Chem.***, 73, 3679-3685, 1969.

[2] Nagata, I., and Hasegawa, T.: Gaseous interdiffusion coefficients, ***J. Chem. Engng. Japan,*** 3, 143-145, 1970.

[3] Getzinger, R. W., and Wilke, C. R.: An experimental study of nonequimolal diffusion in ternary gas mixtures, ***Aiche J.***, 13, 577-580, 1967.

[4] Lugg, G. A.: Diffusion coefficients of some organic and other vapors in air, ***Analytical Chemistry***, 40, 1072-1077, 1968.

[5] Mrazek, R. V., Wicks, C. E., and Prabhu, K. N. S.: Dependence of diffusion coefficient on composition in binary gaseous systems, ***J. Chem. Eng. Data***, 13, 508-510, 1968.

[6] Cowie, M., and Watts, H.: Diffusion of methane and chloromethanes in air, ***Canadian Journal of Chemistry***, 49, 74-77, 1971.

[7] Watts, H.: Temperature dependence of diffusion of carbon tetrachloride, chloroform and methylene chloride vapors in air by a rate of evaporation method, ***Canadian Journal of Chemistry***, 49, 67-73, 1971.

[8] Srivastava, B. N., and Saran, A.: Mutual diffusion studies in krypton-acetone and krypton-chloroform systems, ***Physica***, 32, 110-118, 1966.

[9] Weissman, S.: Estimation of diffusion coefficients from viscosity measurements-polar + polyatomic gases, ***J. Chem. Phys.***, 40, 3397-3406, 1964.

1.8 $CHBr_3$

bath gas	reference	*T*	*P*	D_m	D_e	$(D_m-D_e)/D_e$ (%)
air	[1]	298	760	58±1	71	-18

The measured diffusivity of $CHBr_3$ in air at 298 K [1] is 18% smaller than the estimated value.

References:

[1] Lugg, G. A.: Diffusion coefficients of some organic and other vapors in air, ***Analytical Chemistry***, 40, 1072-1077, 1968.

1.9 CF_2Cl_2

bath gas	reference	T	P	D_m	D_e	$(D_m-D_e)/D_e$ (%)
air	[1]	298	760	72±3	67	7
H_2O	[2]	298	760	80	90	-11
ethanol	[2]	298	760	36	43	-15
benzene	[2]	298	760	29	28	4
di-n-butyl phthalate	[3]	293	760	9.6	13.5	-29
		303	760	10.6	14.3	-26

The differences between the measured diffusivity of CF_2Cl_2 in air,[1] H_2O,[2] ethanol,[2] and benzene [2] at 295 K and estimated values are 7%, 11%, 14%, and 4%, respectively.

Briks et al.[3] measurd the diffusivities of CF_2Cl_2 in di-n-butyl phthalate at 293 and 303 K, and the measured diffusivities are 29% and 26% smaller than the measured values.

References:

[1] Barr, R. F., and Watts, H.: Diffusion of some organic and inorganic compounds in air, ***J. Chem. Eng. Data***, 17, 45-46, 1972.

[2] Lee, C. Y., and Wilke, C. R.: Measurements of vapor diffusion coefficient, ***Industrial and Engineering Chemistry***, 46, 2381-2387, 1954.

[3] Birks, J., and Bradley, R. S.: The rate of evaporation od droplets. 2. the influence of changes of temperature and of the surrounding gas on the rate of evaporation of drops of di-normal-butyl phthalate, ***Proceedings of the Royal Society of London Series a-Mathematical and Physical Sciences***, 198, 226-239, 1949.

1.10 CF_4

bath gas	reference	*T*	*P*	D_m	D_e	$(D_m-D_e)/D_e$ (%)
CH_4	[1]	298	760	92.7	87.6	6
		353	760	126.9	117.5	8
		383	760	145.9	135.5	8
SF_6	[2]	303	760	36.3	32.7	11
		313	760	38.5	34.6	11
		329	760	42.1	37.8	12
		342	760	45.2	40.4	12
n-hexane	[3]	283	760	29.6	26.6	11
		298	760	33.1	29.1	13
		313	760	36.6	31.8	15
		328	760	39.7	34.5	15
cyclohexane	[4]	283	760	30.9	27.1	14
		298	760	33.7	29.7	14
		313	760	37.3	32.4	15
		328	760	40.2	35.1	14
		343	760	43.6	38.0	15
benzene	[4]	283	760	34.7	30.8	13
		298	760	37.5	33.7	11
		313	760	42.1	36.7	15
		328	760	45.4	39.9	14
		343	760	49.2	43.1	14
n-heptane	[3]	283	760	28.5	24.3	17
		298	760	29.4	26.6	11
		313	760	32.3	29.0	11
		328	760	35.7	31.5	14
		343	760	38.3	34.0	13
methylcyclohexane	[4]	283	760	25.9	24.7	5
		298	760	29.2	27.0	8
		313	760	33.7	29.5	14
		328	760	36.1	32.0	13
		343	760	39.2	34.6	13

toluene	[4]	283	760	29.4	27.5	7
		298	760	33.1	30.1	10
		313	760	36.6	32.8	11
		328	760	40.0	35.6	12
		343	760	43.9	38.5	14
octane	[3]	283	760	20.9	22.5	-7
		283	760	24.1	22.5	7
		298	760	27.4	24.6	12
		298	760	27.7	24.6	12
		313	760	31.8	26.8	19
		313	760	29.6	26.8	11
		328	760	33.1	29.1	14
		328	760	32.7	29.1	12
		343	760	35.2	31.5	12
		343	760	34.4	31.5	9

Mueller and Cahill [1] measured the diffusivities of CF_4 in CH_4 from 298-383 K, and the differences between the measured and estimated diffusivities are around 8% or smaller.

Raw and Tang [2] measured the diffusivities of CF_4 in SF_6 from 303 to 342 K, and the differences between the measured and estimated diffusivities are around 12%.

Wilhelm and Battino [3] measured the diffusivities of CF_4 in n-haxane from 283 to 328 K, and the measuered diffusivities are 11% to 15% larger than the estimated values.

Wilhelm et al. [4] measured the diffusivities of CF_4 in cyclohaxane from 283 to 343 K, and the measured diffusivities are ~15% larger than the estimated values.

The diffusivities of CF_4 in benzene are measuered by Wilhelm from 283 to 343 K,[4] and the differences between the measured and estimated diffusivities are around 15% or smaller.

Wilhelm and Battino [3] measured the diffusivities of CF_4 in n-heptane from 283 to 343 K, and the differences between the measured and estimated diffusivities are 17% or smaller.

Wilhelm et al.[4] measured the diffusivities of CF_4 in methylcyclohexane from 283 to 343 K, and the measured diffusivities are 5% to 14% larger than the estimated values.

Wilhelm et al.[4] measured the diffusivities of CF_4 in toluene from 283 to 343 K, and the differences between the measured and estimated values are 14% or smaller.

Wilhelm and Battino [3] measured the diffusivities of CF_4 in octane from 283 to 343 K, and the difference between the measured and estimated diffusivities are 19% or smaller.

References:

[1] Mueller, C. R., and Cahill, R. W.: Mass spectrometric measurement of diffusion coefficients, ***J. Chem. Phys.***, 40, 651-654, 1964.

[2] Raw, C. J. G., and Tang, H.: Viscosity and diffusion coefficients of gaseous sulfur hexafluoride-carbon tetrafluoride mixtures, ***J. Chem. Phys.***, 39, 2616-2618, 1963.

[3] Wilhelm, E., and Battino, R.: Binary gaseous diffusion-coefficients .1. methane and carbon tetrafluoride with hexane, heptane, octane, and 2,2,4-trimethylpentane at one-atmosphere pressure at 10-70 degrees c, ***J. Chem. Eng. Data***, 17, 187-189, 1972.

[4] Wilhelm, E., Battino, R., and Carpente.Rl: Binary gaseous diffusion-coefficients .2. methane and carbon tetrafluoride with cyclohexane, methylcyclohexane, benzene, and toluene at 1atm at 10-70 degrees c, ***J. Chem. Eng. Data***, 19, 245-246, 1974.

1.11 CCl_4

bath gas	reference	T	P	D_m	D_e	$(D_m-D_e)/D_e$ (%)
N_2	[1]	364	760	86	90	-5
		383	760	94	99	-5
		403	760	102	108	-6
		423	760	112	117	-5
	[2]	353	760	84	86	-2
air	[3]	298	760	63±1	62	2
	[4]	298	760	58±1	62	-7
	[5]	308	760	68	65	4
	[6]	295	740	58	61	-5
		296	743	57	61	-6
	[7]	298	760	59	62	-5
		308	760	63	65	-4
		318	760	66	69	-4
		328	760	70	73	-4
		338	760	74	77	-4
		348	760	78	81	-4
	[8]	295	762	61	61	1
	[9]	313	760	67	67	1
CO_2	[1]	363	760	65	68	-5
		384	760	71	75	-6
		403	760	76	82	-7
		423	760	84	89	-5
benzene	[10]	293	760	19±1	17	12
CH_2Cl_2	[10]	293	760	22±1	29	-26
		353	760	32±1	40	-21
		413	760	42±4	53	-22
$CHCl_3$	[10]	293	760	20±1	24	-17

The diffusivities of CCl_4 in N_2 were measured by Nagata and Hasegawa [1] from 364 to 423 K and by Arinikar [2] at 353 K. The measured diffusivities are 2% to 6% smaller than the estimated values.

Five studies measured the diffusivities of CCl_4 in air at 295-298 K,[3, 4, 6-8] and the differences between the measured and estimated diffusivities are 7% or smaller. Getzinger and Wilke [5] and Richardson [9] measured the diffusivities of CCl_4 in air at 308 and 313 K, and the differences between the measured and estimated values are 4% or smaller. The temperature dependence of diffusivities of CCl_4 in air was investigated from 298 to 348 K by Watts.[7] The differences between the measured and estimated diffusivities are 5% or smaller across the entire temperature range investigated.

Nagata and Hasegawa [1] measured the diffusivities of CCl_4 in CO_2 from 363 to 423 K, and the measured diffusivities are around 6% smaller than the estimated values.

Weissman [10] measured the diffusivity of CCl_4 in benzene at 293 K, and the measured diffusivity is 12% larger than the estimated value.

Weissman [10] measured the diffusivities of CCl_4 in CH_2Cl_2 at 293, 353, and 413 K, and the measured diffusivities are all around 25% smaller than estimated values.

The diffusivity of CCl_4 in $CHCl_3$ was measured by Weissman at 293 K,[10] and the measured diffusivity is 17% smaller than the estimated value.

References:

[1] Nagata, I., and Hasegawa, T.: Gaseous interdiffusion coefficients, ***J. Chem. Engng. Japan***, 3, 143-145, 1970.

[2] Arnikar, H. J., Rao, T. S., and Karmarkar, K. H.: Eletrodeless discharge as detector in gas chromatography. 3. study of inter-diffusion of gases, ***International Journal of Electronics***, 22, 381-385, 1967.

[3] Lugg, G. A.: Diffusion coefficients of some organic and other vapors in air, ***Analytical Chemistry***, 40, 1072-1077, 1968.

[4] Cowie, M., and Watts, H.: Diffusion of methane and chloromethanes in air, ***Canadian Journal of Chemistry***, 49, 74-77, 1971.

[5] Getzinger, R. W., and Wilke, C. R.: An experimental study of nonequimolal diffusion in ternary gas mixtures, ***Aiche J.***, 13, 577-580, 1967.

[6] Grob, A. K., and Elwakil, M. M.: An interferometric technique for measuring binary diffusion coefficients, ***Journal of Heat Transfer***, 91, 259-265, 1969.

[7] Watts, H.: Temperature dependence of diffusion of carbon tetrachloride, chloroform and methylene chloride vapors in air by a rate of evaporation method, ***Canadian Journal of Chemistry***, 49, 67-73, 1971.

[8] Pryde, J. A., and Pryde, E. A.: A simple quantitative diffusion experiment, ***Physics Education***, 2, 311-314, 1967.

[9] Richardson, J. F.: The evaporation of two-component liquid mixtures, ***Chem. Eng. Sci.***, 10, 234-242, 1959.

[10] Weissman, S.: Estimation of diffusion coefficients from viscosity measurements-polar + polyatomic gases, ***J. Chem. Phys.***, 40, 3397-3406, 1964.

1.12 CCl_2O, CCl_3NO_2, and CNCl

species	bath gas	reference	T	P	D_m	D_e	$(D_m-D_e)/D_e$ (%)
CCl_2O	air	[1]	273	760	72	66	9
CCl_3NO_2	air	[1]	298	760	67	63	7
		[2]	298	760	62±1	63	-2
CNCl	air	[1]	273	760	84	84	1

The measured diffusivities in air are 9% larger than the estimated value for CCl_2O and 1% larger for CNCl.[1]

Two studies measured the diffusivities of CCl_3NO_2 in air at 298 K, and the measured values are 7% larger [1] and 2% smaller [2] than the estimated ones.

References:

[1] Klotz, I. M., and Miller, D. K.: Diffusion coefficients and molecular radii of hydrogen cyanide, cyanogen chloride, phosgene and chloropicrin, J. Am. Chem. Soc., 69, 2557-2558, 1947.

[2] Lugg, G. A.: Diffusion coefficients of some organic and other vapors in air, Analytical Chemistry, 40, 1072-1077, 1968.

2 Compounds with two carbon atoms

2.1 CH_3CH_2Cl

bath gas	reference	T	P	D_m	D_e	$(D_m-D_e)/D_e$ (%)
CH_3Cl	[1]	298	731	43	54	-22
		298	736	42	55	-22
		358	742	64	75	-14
		378	741	74	83	-10
		419	741	90	99	-9
		438	731	98	107	-9
Kr	[2]	275	21	45	50	-9
		288	28	55	54	2
		303	21	54	59	-7
		318	24	60	64	-6

Gotoh et al.[1] measured the diffusivities in CH_3Cl from 298 to 438 K, and the differences between the measured and estimated diffusivities are around 22% or less.

The diffusivities of CH_3CH_2Cl in Kr were measured from 275 to 318 K by Singh and Srivastava,[2] and the differences between the measured and estimated diffusivitiies are smaller than 10%.

References:

[1] Gotoh, S., Manner, M., Sorensen, J. P., and Stewart, W. E.: Binary diffusion-coefficients of low-density gases .1. Measurements by modifide loschmidt method, ***J. Chem. Eng. Data***, 19, 169-171, 1974.

[2] Singh, Y., and Srivastava, B. N.: Unlike interactions and binary diffusion in polar-nonpolar mixtures-krypton-methylene chloride and krypton-ethylchloride, ***International Journal of Heat and Mass Transfer***, 11, 1771-1778, 1968.

2.2 CH_3CH_2Br

bath gas	reference	T	P	D_m	D_e	$(D_m-D_e)/D_e$ (%)
He	[1]	428	760	562±8	548	3
Air	[2]	298	760	75±1	84	-11
	[3]	295	745	80	83	-4
		295	729	79	83	-5
		296	736	78	83	-6

The diffusivity of CH_3CH_2Br in He at 428 K, measured by Fuller et al.,[1] is 3% larger than the estimated value.

Lugg [2] and Grob and Elwakil [3] measurd the diffusivities of CH_3CH_2Br in air at around room temperature, and the differences between measured and estimated diffusivities are 4% to 6%.

References:

[1] Fuller, E. N., Ensley, K., and Giddings, J. C.: Diffusion of halogenated hydrocarbons in helium. Effect of structure on collision cross sections, ***J. Phys. Chem.***, 73, 3679-3685, 1969.

[2] Lugg, G. A.: Diffusion coefficients of some organic and other vapors in air, ***Analytical Chemistry***, 40, 1072-1077, 1968.

[3] Grob, A. K., and Elwakil, M. M.: An interferometric technique for measuring binary diffusion coefficients, ***Journal of Heat Transfer***, 91, 259-265, 1969.

2.3 CH_3CH_2I

bath gas	reference	T	P	Dm	De	(Dm-De)/De
air	[1]	295	736	72	69	4
		295	730	72	69	4
		295	737	71	69	3
He	[2]	428	760	492±10	506	-3

The average diffusivity of CH_3CH_2I in air at 295 K, measured by Grob and Elwakil,[1] is ~3% larger then the estimated value.

The diffusivity of CH_3CH_2I in He at 428 K, measured by Fuller et al.,[2] is 3% smaller than the estimated value.

References:

[1] Grob, A. K., and Elwakil, M. M.: An interferometric technique for measuring binary diffusion coefficients, ***Journal of Heat Transfer***, 91, 259-265, 1969.

[2] Fuller, E. N., Ensley, K., and Giddings, J. C.: Diffusion of halogenated hydrocarbons in helium. Effect of structure on collision cross sections, ***J. Phys. Chem.***, 73, 3679-3685, 1969.

2.4 CH_3CHF_2

bath gas	reference	*T*	*P*	D_m	D_e	$(D_m-D_e)/D_e$ (%)
He	[1]	430	760	573±11	527	9

The diffusivity in He at 430 K, measured by Fuller et al.,[1] is 9% larger than the estimated value.

References:

[1] Fuller, E. N., Ensley, K., and Giddings, J. C.: Diffusion of halogenated hydrocarbons in helium. Effect of structure on collision cross sections, ***J. Phys. Chem.***, 73, 3679-3685, 1969.

2.5 CH_2ClCH_2Cl

bath gas	reference	T	P	D_m	D_e	$(D_m-D_e)/D_e$ (%)
He	[1]	427	760	519±5	476	9
air	[2]	295	745	67	68	-2
		295	738	67	68	-1
		295	742	66	68	-4
	[3]	298	760	70±1	70	0
		298	760	69±1	70	-1

The diffusivity of CH_2ClCH_2Cl in He at 427 K, measured by Fuller et al.,[1] is 9% larger than the estimated value.

The diffusivities of CH_2ClCH_2Cl in air were measured by two studies at around room temperature,[2,3] and the differences between the measured and estimated values are 4% or less.

References:

[1] Fuller, E. N., Ensley, K., and Giddings, J. C.: Diffusion of halogenated hydrocarbons in helium. Effect of structure on collision cross sections, ***J. Phys. Chem.***, 73, 3679-3685, 1969.

[2] Grob, A. K., and Elwakil, M. M.: An interferometric technique for measuring binary diffusion coefficients, ***Journal of Heat Transfer***, 91, 259-265, 1969.

[3] Lugg, G. A.: Diffusion coefficients of some organic and other vapors in air, ***Analytical Chemistry***, 40, 1072-1077, 1968.

2.6 CH_2BrCH_2Br

bath gas	reference	*T*	*P*	D_m	D_e	(D_m-De)/D_e (%)
air	[1]	298	760	63±1	72	-13

The diffusivity of CH_2BrCH_2Br in air at 298 K, measured by Lugg,[1] is 13% smaller than the estimated value.

References:

[1] Lugg, G. A.: Diffusion coefficients of some organic and other vapors in air, ***Analytical Chemistry***, 40, 1072-1077, 1968.

2.7 CCl_3CH_3 and $CHCl_2CH_2Cl$

species	reference	bath gas	T	P	D_m	D_e	$(D_m-D_e)/D_e$ (%)
CCl_3CH_3	[1]	air	298	760	60±1	62	-2
$CHCl_2CH_2Cl$	[1]	air	298	760	60±1	62	-2

Lugg [1] measured the diffusivities of CCl_3CH_3 and $CHCl_2CH_2Cl$ in air at 298 K, and the measured diffusivities are both 2% smaller than the estimated values.

References:

[1] Lugg, G. A.: Diffusion coefficients of some organic and other vapors in air, ***Analytical Chemistry***, 40, 1072-1077, 1968.

2.8 $CHCl_2CHCl_2$, CCl_3CHCl_2, and CH_2ClCH_2OH

species	bath gas	reference	*T*	*P*	D_m	D_e	$(D_m-D_e)/D_e$ (%)
$CHCl_2CHCl_2$	air	[1]	298	760	55±1	57	-2
CCl_3CHCl_2	air	[1]	298	760	51±1	53	-2
CH_2ClCH_2OH	air	[1]	298	760	73±1	76	-2

Lugg [1] measured the diffusivities of $CHCl_2CHCl_2$, CCl_3CHCl_2, and CH_2ClCH_2OH in air at 298 K, and the measured diffusivities are all 2% smaller than the estimated values.

References:

[1] Lugg, G. A.: Diffusion coefficients of some organic and other vapors in air, ***Analytical Chemistry***, 40, 1072-1077, 1968.

2.9 CH_2=CHCl, CHCl=CHCl, CHCl=CCl_2, and CCl_2=CCl_2

formula	bath gas	reference	*T*	*P*	D_m	D_e	$(D_m-D_e)/D_e$ (%)
CH_2=CHCl	air	[1]	298	760	93±4	85	-2
CHCl=CHCl	air	[1]	298	760	87±7	71	-2
CHCl=CCl_2	air	[2]	298	760	67±1	63	-2
CCl_2=CCl_2	air	[2]	298	760	61±1	58	-2

Barr and Watts [1] measured the diffusivities of CH_2=CHCl and CHCl=CHCl in air at 298 K, and Lugg [2] measured the diffusivities of CHCl=CCl_2 and CCl_2=CCl_2 in air at 298 K. The measured diffusivities are all 2% smaller than the estimated values.

References:

[1] Barr, R. F., and Watts, H.: Diffusion of some organic and inorganic compounds in air, ***J. Chem. Eng. Data***, 17, 45-46, 1972.

[2] Lugg, G. A.: Diffusion coefficients of some organic and other vapors in air, ***Analytical Chemistry***, 40, 1072-1077, 1968.

3 Compounds with three or more carbon atoms

3.1 $CH_3CH_2CH_2Cl$, $CH_3CH_2CH_2Br$, and $CH_3CHBrCH_3$

species	bath gas	reference	T	P	D_m	D_e	$(D_m-D_e)/D_e$ (%)
$CH_3CH_2CH_2Cl$	He	[1]	428	760	480±7	474	1
$CH_3CH_2CH_2Br$	He	[1]	428	760	450±7	475	-5
	air	[2]	298	760	67±1	73	-8
$CH_3CHBrCH_3$	He	[1]	428	760	461±9	474	-3
	air	[2]	298	760	69±1	73	-4

The measured diffusivity of $CH_3CH_2CH_2Cl$ in He at 428 K [1] is only 1% larger than the estimated value.

The measured diffusivities of $CH_3CH_2CH_2Br$ in He at 428 K [1] and in air at 298 K [2] are 5% and 8% smaller than the estimated values, respectively.

The measured diffusivities of $CH_3CHBrCH_3$ in He at 428 K [1] and in air at 298 K [2] are 3% and 4% smaller than the estimated values, respectively.

References:

[1] Fuller, E. N., Ensley, K., and Giddings, J. C.: Diffusion of halogenated hydrocarbons in helium. Effect of structure on collision cross sections, ***J. Phys. Chem.***, 73, 3679-3685, 1969.

[2] Lugg, G. A.: Diffusion coefficients of some organic and other vapors in air, ***Analytical Chemistry***, 40, 1072-1077, 1968.

3.2 $CH_3CH_2CH_2I$ and CH_3CHICH_3

species	bath gas	reference	*T*	*P*	D_m	D_e	$(D_m\text{-}D_e)/D_e$ (%)
$CH_3CH_2CH_2I$	He	[1]	430	760	440±5	449	-2
	H_2	[2]	303	760	258	297	-13
	N_2	[2]	303	760	60	67	-11
	air	[3]	298	760	66±1	63	5
CH_3CHICH_3	He	[1]	430	760	440±9	449	-2
	air	[3]	298	760	67±1	63	6

The measured diffusivities of $CH_3CH_2CH_2I$ in He at 430 K,[1] in H_2 at 303 K,[2] in N_2 at 303 K,[2] and in air at 298 K [3] are 2% smaller, 13% smaller, 11% smaller, and 5% larger than the estimated values.

The measured diffusivities of CH_3CHICH_3 in He at 430 K [1] and in air at 298 K [3] are 2% smaller and 6% larger than the estimated values.

References:

[1] Fuller, E. N., Ensley, K., and Giddings, J. C.: Diffusion of halogenated hydrocarbons in helium. Effect of structure on collision cross sections, ***J. Phys. Chem.***, 73, 3679-3685, 1969.

[2] Byrne, J. J., Maguire, D., and Clarke, J. K. A.: Gas-phase interdiffusion coefficients for some polar organic compounds, ***J. Phys. Chem.***, 71, 3051-3052, 1967.

[3] Lugg, G. A.: Diffusion coefficients of some organic and other vapors in air, ***Analytical Chemistry***, 40, 1072-1077, 1968.

3.3 $CH_3CHClCH_2Cl$, $CH_3CHBrCH2Cl$, $CH_3BrCHBrCH_2Cl$, and $CH_2=CHCH_2Cl$

species	bath gas	reference	T	P	D_m	D_e	$(D_m-D_e)/D_e$ (%)
$CH_3CHClCH_2Cl$	air	[1]	298	760	60±1	63	-3
$CH_3CHBrCH_2Cl$	He	[2]	427	760	433±12	423	2
$CH_2BrCHBrCH_2Cl$	air	[1]	298	760	52±1	58	-9
$CH_2=CHCH_2Cl$	air	[1]	298	760	74±1	73	2

The measured diffusivities in air at 298 K [1] are 3% smaller than the estimated value for CH_3CHCH_2Cl, 9% smaller for $CH_3CHBrCH_2Cl$, and 2% larger for $CH_2=CHCH_2Cl$, respectively.

The measured diffusivity of $CH_3CHBrCH_2Cl$ in He at 427 K [2] is 2% larger than the estimated value.

References:

[1] Lugg, G. A.: Diffusion coefficients of some organic and other vapors in air, ***Analytical Chemistry***, 40, 1072-1077, 1968.

[2] Fuller, E. N., Ensley, K., and Giddings, J. C.: Diffusion of halogenated hydrocarbons in helium. Effect of structure on collision cross sections, ***J. Phys. Chem.***, 73, 3679-3685, 1969.

3.4 1-chlorobutane and 2-chlorobutane

species	bath gas	reference	T	P	D_m	D_e	$(D_m-D_e)/D_e$ (%)
1-chlorobutane	He	[1]	429	760	422±8	425	-1
	air	[2]	296	744	67±1	63	8
			296	743	68	63	8
			295	748	67	62	7
2-chlorobutane	He	[1]	429	760	426±6	425	0

The differences between the measured and estimated diffusivities in He at 429 K are around 1% for both 1-chlorobutane and 2-chlorobutane.[1]

The average measured diffusivity of 1-chlorobutane in air at 296 K [2] is 8% larger than the estimated value.

References:

[1] Fuller, E. N., Ensley, K., and Giddings, J. C.: Diffusion of halogenated hydrocarbons in helium. Effect of structure on collision cross sections, J. Phys. Chem., 73, 3679-3685, 1969.

[2] Grob, A. K., and Elwakil, M. M.: An interferometric technique for measuring binary diffusion coefficients, Journal of Heat Transfer, 91, 259-265, 1969.

3.5 1–bromobutane and 2-bromobutane

species	bath gas	reference	T	P	D_m	D_e	$(D_m-D_e)/D_e$ (%)
1-bromobutane	He	[1]	427	760	414±5	421	-2
	H_2	[2]	303	760	253	281	-10
	N_2	[2]	303	760	61	68	-10
2-bromobutane	He	[1]	427	760	420±10	422	-1

The measured diffusivities of 1-bromobutane in He at 427 K,[1] in H_2 at 303 K,[2] and in N_2 at 303 K [2] are 2%, 10%, and 10% smaller than the estimated values.

The difference between the measured [1] and estimated diffusivities of 2-bromobutane in He at 427 K is less than 1%.

References:

[1] Fuller, E. N., Ensley, K., and Giddings, J. C.: Diffusion of halogenated hydrocarbons in helium. Effect of structure on collision cross sections, ***J. Phys. Chem.***, 73, 3679-3685, 1969.

[2] Byrne, J. J., Maguire, D., and Clarke, J. K. A.: Gas-phase interdiffusion coefficients for some polar organic compounds, ***J. Phys. Chem.***, 71, 3051-3052, 1967.

3.6 1-iodobutane, 2-iodobutane, and dichloroethyl ether

Species	bath gas	reference	*T*	*P*	D_m	D_e	$(D_m-D_e)/D_e$ (%)
1-iodobutane	He	[1]	428	760	398±5	402	-1
2-iodobutane	He	[1]	427	760	414±10	400	3
dichloroethyl ether	air	[2]	298	760	53	55	-5

The differences between the measured and estimated diffusivities are 1% for 1-iodobutane in He at 428 K,[1] 3% for 2-iodobutane in He at 427 K,[1] and 5% for dichloroethyl ether in air at 298 K,[2] respectively.

References:

[1] Fuller, E. N., Ensley, K., and Giddings, J. C.: Diffusion of halogenated hydrocarbons in helium. Effect of structure on collision cross sections, ***J. Phys. Chem.***, 73, 3679-3685, 1969.

[2] Lugg, G. A.: Diffusion coefficients of some organic and other vapors in air, ***Analytical Chemistry***, 40, 1072-1077, 1968.

3.7 1-chloropentane and 1-bromo-3-methyl-butane

species	bath gas	reference	T	P	D_m	D_e	$(D_m-D_e)/D_e$ (%)
1-chloropentane	He	[1]	428	760	394±3	385	2
1-bromo-3-methyl-butane	H_2	[2]	303	760	235	258	-9
	N_2	[2]	303	760	61	62	-2
	Ar	[2]	303	760	50	56	-11

The measured diffusivity of 1-chloropentane in He at 428 K [1] is 2% larger than the estimated value.

Byrne et al.[2] measured the diffusivities of 1-bromo-3-methyl-butane at 303 K. The differences between measurements and estimations are 9%, 2%, and 11% for diffusivities in H_2, N_2, and Ar, respectively.

References:

[1] Fuller, E. N., Ensley, K., and Giddings, J. C.: Diffusion of halogenated hydrocarbons in helium. Effect of structure on collision cross sections, ***J. Phys. Chem.***, 73, 3679-3685, 1969.

[2] Byrne, J. J., Maguire, D., and Clarke, J. K. A.: Gas-phase interdiffusion coefficients for some polar organic compounds, ***J. Phys. Chem.***, 71, 3051-3052, 1967.

3.8 1-fluorohexane, 1-bromohexane, 2-bromohexane, and 3-bromohexane

species	bath gas	reference	*T*	*P*	D_m	D_e	$(D_m-D_e)/D_e$ (%)
1-fluorohexane	He	[1]	432	760	374±5	369	1
1-bromohexane	He	[1]	428	760	350±6	354	-1
2-bromohexane	He	[1]	428	760	357±9	354	1
3-bromohexane	He	[1]	429	760	356±33	355	1

Fuller et al.[1] measured the diffusivities of 1-fluorohexane, 1-bromohexane, 2-bromohexane, and 3-bromohexane in He at ~430 K. The differences between the measured and estimated diffusivities are around 1% for all the four compounds.

References:

[1] Fuller, E. N., Ensley, K., and Giddings, J. C.: Diffusion of halogenated hydrocarbons in helium. Effect of structure on collision cross sections, ***J. Phys. Chem.***, 73, 3679-3685, 1969.

3.9 C_7F_{18}

bath gas	reference	*T*	*P*	D_m	D_e	$(D_m-D_e)/D_e$ (%)
C_8H_{18}	[1]	303	760	9.0±0.2	11.0	-18
		323	760	10.2±0.5	12.3	-17

The measured diffusivities of C_7F_{18} in C_8H_{18} [1] are 18% smaller than the estimated value at 303 K and 17% smaller at 323 K.

References:

[1] Weissman, S.: Estimation of diffusion coefficients from viscosity measurements-polar + polyatomic gases, ***J. Chem. Phys.***, 40, 3397-3406, 1964.

4 Aromatic compounds

4.1 fluorobenzene, chlorobenzene, and bromobenzene

species	bath gas	reference	T	P	D_m	D_e	$(D_m-D_e)/D_e$ (%)
fluorobenzene	He	[1]	430	760	430±6	395	9
	H_2	[2]	303	760	287	263	9
	N_2	[2]	303	760	70	63	11
	air	[3]	295	734	61	58	4
		[3]	295	729	61	58	5
		[3]	295	735	61	58	4
	Ar	[2]	303	760	63	57	10
chlorobenzene	He	[1]	431	760	412±5	386	7
	air	[4]	299	760	56	57	-2
		[4]	313	760	60	62	-3
		[4]	332	760	68	69	-1
		[5]	298	760	57	57	-1
bromobenzene	He	[1]	427	760	413±8	379	9

The diffusivity of fluorobenzene in He at 430 K was measured by Fuller et al.,[1] and the measured diffusivity is 9% larger than the estimated value.

The diffusivity of fluorobenzene in H_2 at 303 K was measured by Byrne et al.,[2] and the measured diffusivity is 9% larger than the estimated value.

The diffusivity of fluorobenzene in Ar at 303 K was measured by Byrne et al.,[2] and the measured diffusivity is 10% larger than the estimated value.

The diffusivity of fluorobenzene in N2 at 303 K, measured by Byrne et al.,[2] is 11% larger than the estimated value.

The average diffusivity of fluorobenzene in N2 at 295 K, measured by Grob and Elwakil,[3] is ~5% larger than the estimated value.

Fuller et al.[1] measured the diffusivity of chlorobenzene in He, and the measured diffusivity is 7% larger than the estimated value.

The diffusivities of chlorobenzene in air were measured by two studies.[4, 5] The diffusivities from 299 to 332 K, measured by Gilliland,[4] are around 3% (or less) smaller than the estimated values, and the diffusivity at 298 K, measured by Lugg,[5] is only 1% smaller than the estimated value.

The diffusivity of bromobenzene in He, measured by Fuller et al.,[1] is 9% larger than the estimated value.

References:

[1] Fuller, E. N., Ensley, K., and Giddings, J. C.: Diffusion of halogenated hydrocarbons in helium. Effect of structure on collision cross sections, ***J. Phys. Chem.***, 73, 3679-3685, 1969.

[2] Byrne, J. J., Maguire, D., and Clarke, J. K. A.: Gas-phase interdiffusion coefficients for some polar organic compounds, ***J. Phys. Chem.***, 71, 3051-3052, 1967.

[3] Grob, A. K., and Elwakil, M. M.: An interferometric technique for measuring binary diffusion coefficients, Journal of Heat Transfer, 91, 259-265, 1969.

[4] Gilliland, E. R.: Diffusion coefficients in gaseous systems, ***Industrial and Engineering Chemistry***, 26, 681-685, 1934.

[5] Lugg, G. A.: Diffusion coefficients of some organic and other vapors in air, ***Analytical Chemistry***, 40, 1072-1077, 1968.

4.2 hexafluorobenzene

bath gas	reference	*T*	*P*	D_m	D_e	$(D_m\text{-}D_e)/D_e$ (%)
He	[1]	429	760	344±6	314	10

The diffusivity of hexafluorobenzene in He at 429 K, measured by Fuller et al.,[1] is 10% larger than the estimated value.

References:

[1] Fuller, E. N., Ensley, K., and Giddings, J. C.: Diffusion of halogenated hydrocarbons in helium. Effect of structure on collision cross sections, ***J. Phys. Chem.***, 73, 3679-3685, 1969.

4.3 4-fluorotoluene, 2-chlorotoluene, 3-chlorotoluene, 4-chlorotoluene, and benzyl chloride

species	bath gas	reference	T	P	D_m	D_e	$(D_m-D_e)/D_e$ (%)
4-fluorotoluene	He	[1]	432	760	386±5	366	5
2-chlorotoluene	air	[2]	298	760	52	53	-1
3-chlorotoluene	air	[2]	298	760	49	53	-7
4-chlorotoluene	air	[2]	298	760	47	53	-11
benzyl chloride	air	[2]	298	760	54	53	2

The diffusivity of 4-fluorotoluene in He at 432 K, measured by Fuller et al.,[1] is 5% larger than the estimated value.

Lugg [2] measured the diffusivities of 2-chlorotoluene, 3-chlorotoluene, 4-chlorotoluene, and benzyl chloride in air at 298 K. The differences between the measured and estimated diffusivities are 1%, 7%, 11%, and 2% for 2-chlorotoluene, 3-chlorotoluene, 4-chlorotoluene, and benzyl chloride, respectively.

References:

[1] Fuller, E. N., Ensley, K., and Giddings, J. C.: Diffusion of halogenated hydrocarbons in helium. Effect of structure on collision cross sections, ***J. Phys. Chem.***, 73, 3679-3685, 1969.

[2] Lugg, G. A.: Diffusion coefficients of some organic and other vapors in air, ***Analytical Chemistry***, 40, 1072-1077, 1968.
